# Femtosecond Laser Spectroscopy of the Rhodopsin Photochromic Reaction: A Concept for Ultrafast Optical Molecular Switch Creation (Ultrafast Reversible Photoreaction of Rhodopsin)

**DOI:** 10.3390/molecules191118351

**Published:** 2014-11-11

**Authors:** Olga Smitienko, Victor Nadtochenko, Tatiana Feldman, Maria Balatskaya, Ivan Shelaev, Fedor Gostev, Oleg Sarkisov, Mikhail Ostrovsky

**Affiliations:** 1 Emanuel Institute of Biochemical Physics, Russian Academy of Sciences, Kosygin st.4, Moscow 119334, Russia; E-Mails: djolia@gmail.com (O.S.); nadtochenko@gmail.com (V.N.); ostrovsky3535@mail.ru (M.O.); 2 Semenov Institute of Chemical Physics, Russian Academy of Sciences, Kosygin st.4, Moscow 119991, Russia; E-Mails: shelaev@bk.ru (I.S.); boatsween@yandex.ru (F.G.); sarkisov@femto.chph.ras.ru (O.S.); 3 Institute of Problems of Chemical Physics, Russian Academy of Sciences, Academician Semenov avenue 1, Chernogolovka, Moscow region 142432, Russia; 4 Biological Faculty, Lomonosov Moscow State University, Leninskie Gory 1, Moscow 119991, Russia; 5 Faculty of Basic Medicine, Lomonosov Moscow State University, Lomonosovsky pr. 31/5, Moscow 119192, Russia; E-Mail: m.balatskaya@gmail.com

**Keywords:** rhodopsin, femtosecond spectroscopy, coherent reaction, photoreversibility, molecular switches

## Abstract

Ultrafast reverse photoreaction of visual pigment rhodopsin in the femtosecond time range at room temperature is demonstrated. Femtosecond two-pump probe experiments with a time resolution of 25 fs have been performed. The first pump pulse at 500 nm initiated *cis-trans* photoisomerization of rhodopsin chromophore, 11-*cis* retinal, which resulted in the formation of the primary ground-state photoproduct within a mere 200 fs. The second pump pulse at 620 nm with a varying delay of 200 to 3750 fs relative to the first pump pulse, initiated the reverse phototransition of the primary photoproduct to rhodopsin. The results of this photoconversion have been observed on the differential spectra obtained after the action of two pump pulses at a time delay of 100 ps*.* It was found that optical density decreased at 560 nm in the spectral region of bathorhodopsin absorption and increased at 480 nm, where rhodopsin absorbs. Rhodopsin photoswitching efficiency shows oscillations as a function of the time delay between two pump pulses. The quantum yield of reverse photoreaction initiated by the second pump pulse falls within the range 15% ± 1%. The molecular mechanism of the ultrafast reversible photoreaction of visual pigment rhodopsin may be used as a concept for the development of an ultrafast optical molecular switch.

## 1. Introduction

Retinal-binding proteins form a group of photosensitive transmembrane proteins capable of converting the energy of light to perform biological functions of the living organisms. This group comprises sensor rhodopsins that provide light perception as an information carrier (visual pigments of animals, sensor rhodopsins of microorganisms) and those performing photovoltaic energetic function, targeting ion transport through the cellular membrane (e.g., halorhodopsin, bacteriorhodopsin) [1,2].

These pigment-protein complexes have a similar spatial organization and common chromophore group: the retinal (vitamin A aldehyde) residue, which is covalently bound to protein through a protonated Schiff base linkage to a lysine residue from the polypeptide chain of protein [3,4]. Retinal-binding proteins possess unique photochemical properties. In these natural pigment-protein complexes, the chromophore photoisomerization rate shows a femtosecond time range [5,6,7,8,9,10,11]. Therefore, ever-increasing interest in the detailed study of photochemical transformation mechanisms of retinal-binding proteins is observed for the purpose of using the functional principles of these natural photosensors in the creation of fast response devices [12,13,14,15,16,17].

Bovine rhodopsin is one of the well-studied representatives of retinal-binding proteins. It is a typical member of the G-protein-coupled receptor family. The rhodopsin molecule consists of the opsin protein and a chromophore, 11-*cis* retinal. Light quantum absorption leads to 11-*cis* retinal isomerization to the all-*trans* form. Chromophore photoisomerization provides conformational changes in the protein part of the molecule, resulting in the formation of intermediates with various lifetimes and spectral properties. Finally, rhodopsin phototransformation (photolysis) results in the hydrolysis of the Schiff base linkage and all-*trans* retinal release from the rhodopsin molecule.

The elementary act of chromophore photoisomerization in rhodopsin proceeds during a time of about 80–100 fs [7,11,18,19,20] with a quantum yield of 0.65 [21]. Meanwhile, almost all of the energy of the light quantum absorbed (>60%) is converted to chemical energy conformational protein rearrangements [22]. Retinal obtains unique photochemical properties of this kind due to its protein environment. In femtosecond spectroscopic experiments with a time resolution of several tens of femtoseconds, during the initial ~1.5 picoseconds on the absorption kinetic curves of primary rhodopsin photoproduct, oscillations provided by the formation of vibrationally coherent wave packets can be observed [7,8,9,18,23]. Fast Fourier transform analysis of these oscillations allowed the determination of constituent vibrational frequencies of these wave packets. Femtosecond-stimulated Raman spectroscopy methods [24] and theoretical research [19] have detected high-frequency vibrational modes participating in rhodopsin chromophore photoisomerization, C=C (1548 cm^−1^), C-C and C-H (1100–1300 cm^−1^) and HOOP (969 cm^−1^) [24]. They also indicated the extreme importance of HOOP oscillations in the reaction. The pump degenerate four-wave mixing (pump-DFWM) method applied to the study of the excited state of all-*trans* retinal Schiff bases [25] provided a frequency (~1700 cm^−1^) assigned to C=N oscillations that argued for the participation of this high-frequency mode in retinal photoisomerization in rhodopsin.

All rhodopsin photolysis intermediates possess photochromic properties. Absorption of the second light quantum by the photolysis intermediate may cause reverse *trans*-*cis* isomerization of retinal [26,27]. In this case, the formation of rhodopsin with 11-*cis* retinal (photoregeneration process) with an insignificant admixture of isorhodopsin with 9-*cis* retinal occurs [26].

The photochromism of rhodopsin, as well as all other retinal-binding proteins, *i.e.*, the ability to exist in two or more stable (quasi-stable) forms, between which reversible phototransitions are possible, allows the consideration of these pigment-protein complexes to be prototypes of optical molecular switches [12,13,14,16,28,29]. For that matter, the early stages of retinal-binding protein phototransformations are the most attractive. Photochromism in bacteriorhodopsin has found use in the creation of photosensitive media in optical devices for information writing, transmission and storage, with a typical switching time of about milliseconds or nanoseconds [30,31,32]. Yan *et al.* [33] has described primary experiments on ultrafast reversible photoswitching of bovine rhodopsin affected by femtosecond (300 fs) laser pulses.

Coherent control of retinal photoisomerization in bacteriorhodopsin was performed with the shaped femtosecond pulse [34], which provided the modulation of the quantum yield of the *all-trans* retinal→13-*cis* retinal photoreaction by ±20%.

In this work, an approach was applied that used two femtosecond pump pulses (500 nm, 25 fs and 620 nm, 30 fs) following one another with different time delays. The first pump pulse of 500 nm activated direct rhodopsin photoreaction, and the second, delayed pump pulse of 620 nm excited the first photoproduct at different phases of its absorption oscillation. In this case, the dynamics of the wave packet in the first photoproduct can affect the efficiency of the reverse photoreaction of rhodopsin. Then, in particular, the second pump pulse may be the controlling one, due to the timing with the coherent wave packet phase of the first photoproduct of rhodopsin.

The goal of this work was to study the photochromic transition of primary intermediates of bovine rhodopsin photolysis in the femto- and pico-second time range. Another goal was to demonstrate the possibility of the coherent control of reverse rhodopsin photoreaction efficiency via timing the arrival of the second pump pulse with the wave packet phase in the first photoproduct of the direct reaction. In the work, the efficiency dependence for such a photochromic transition on the time delay between the first and the second pump pulses was demonstrated.

## 2. Results and Discussion

### 2.1. Photo-Induced Rhodopsin Transformation to Primary Ground-State Rhodopsin Photoproduct

**Figure 1:**
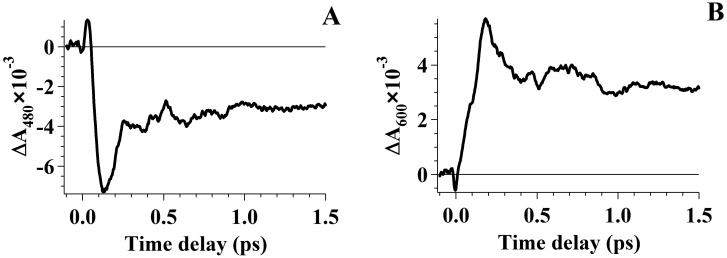
Transient photo-induced absorption kinetic curves of rhodopsin. The kinetic curves were documented by one-pump probe experiments with pumping by a 25 fs pulse at 500 nm and probing at 480 nm (**A**) and 600 nm (**B**).

The photoexcitation of rhodopsin (Rh_498_) by a 25 fs pump pulse at 500 nm results in 11-*cis* retinal chromophore photoisomerization to the *trans*-form and formation of primary ground-state rhodopsin photoproduct (Photo_570_) by the 200th fs that, following picosecond vibrational relaxation, leading to the production of the metastable intermediate, bathorhodopsin (Batho_535_). Figure 1 shows the kinetic curves of photo-induced Rh_498_ absorption at 480 nm (A) and at 600 nm (B).

At a probe wavelength of 480 nm (Figure 1A) at the earliest times (up to 50 fs), a low, positive absorption associated with the excited state absorption of the rhodopsin molecule (S_1_→S_n_) is observed [7,8,9]. At times up to 130 fs, negative absorption, *i.e.*, bleaching of Rh_498_, is observed. Thereafter, during several picoseconds, partial absorption recovery, due to the transition of a portion of excited molecules to the initial state of Rh_498_, and Photo_570_ formation and its further relaxation to Batho_535_, which also absorb in this spectral range, are observed. At a probe wavelength of 600 nm (Figure 1B), Photo_570_ formation may be observed, which completes by 200th fs after photoexcitation.

Irrespective of the probe wavelength, for all kinetic curves, oscillation processes were observed. Our earlier works [9,12,35] show the results of Fourier analysis, which prove the presence of oscillations in the dynamics of photo-induced absorption by reaction products. At times of 0.9–1 ps, the frequencies of the vibrational modes comprised by wave packets of Photo_570_, Batho_535_ and Rh_498_ were determined. In the entire Photo_570_ and Batho_535_ absorption band, the same set of frequencies was obtained—59, 117, 156, 234, 254 and 351 cm^−1^—with notable predominance of the 59 cm^−1^ frequency, whereas in the Rh_498_ absorption band, a similar set of frequencies with slightly lower values was obtained—39, 59, 136, 195, 234 and 371 cm^−1^—with the predominance of the 136 cm^−1^ frequency. Detected oscillation frequencies correspond to vibrational modes, which are active within the elementary act of 11-*cis* retinal isomerization and during the recovery of Rh_498_ [9,35]. It is worth noting that by 3 ps, approximately, the oscillation relaxation process has already completed, and no oscillations were observed.

### 2.2. Photo-Induced Transition of Primary Ground-State Rhodopsin Photoproduct to Rhodopsin

**Figure 2:**
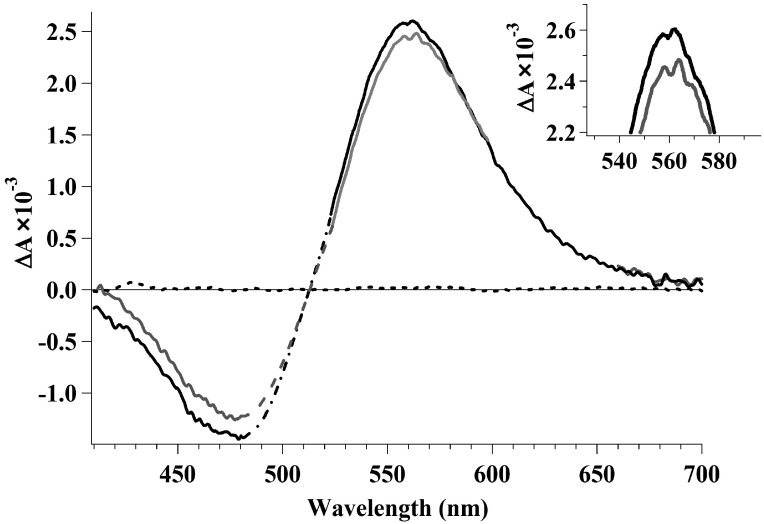
Differential spectra of photo-induced rhodopsin absorption obtained under the effect of a one-pump pulse of 500 nm (*pump1*) (black curve) and two pump pulses of 500 and 620 nm (*pump1* and *pump2*) (grey curve) that follow with a 200-fs time delay and with a probe pulse time delay of 100 ps. For the control, a similar spectrum of completely bleaching rhodopsin affected by a single pump pulse of 500 nm (dashed curve) was registered. In the spectral regions of pump pulses, experimental curves were completed. *Insert*: expanded part of the Batho_535_ maximum absorption area.

For reverse photoreaction of Photo_570_ or Batho_535_ transformation to Rh_498_, a femtosecond two-pump probe pulse setup with two pump pulses (hereinafter referred to as *pump1* and *pump2*) and a supercontinuum probing pulse were used (see “Materials and Methods”). The twenty five-femtosecond *pump1* at 500 nm initiates the reaction 11-*cis retinal* + hv_1_ → *trans*-retinal, and by the 200th fs, Photo_570_ is formed, which relaxes to Batho_535_ with a characteristic time of 1–3 ps. Differential spectra showing the dynamics of Batho_535_ formation and Rh_498_ disappearing in 100 ps after *pump1* action in the spectral range of 410–700 nm were registered (Figure 2, black curve). The thirty-femtosecond *pump2* at 620 nm was supplied after the 25-fs *pump1* at 500 nm with a time delay from hundreds of femtoseconds to several picoseconds. The probe pulse time delay relative to *pump1* was 100 ps. Figure 2 (grey curve) shows the results of two pump pulses affecting the sample and following one another with a 200-fs time delay, *i.e*., in this experiment, *pump2* affected Photo_570_. By the 100th ps after the consecutive action of *pump1* and *pump2*, a decrease of Batho_535_ dark formation and a simultaneous increase of initial Rh_498_ absorption was observed. This result means that *pump2* induces a photoreaction, in which Photo_570_ transforms back to Rh_498_. To put it differently, the reverse *trans-cis* isomerization reaction of retinal residue occurs: *trans*-retinal+hv_2_→11-*cis* retinal. It is worth noting that after the effect of *pump2*, the form of differential spectra did not changed. This may suggest that reverse phototransition Photo_570_**→**Rh_498_ gives no side products.

**Figure 3:**
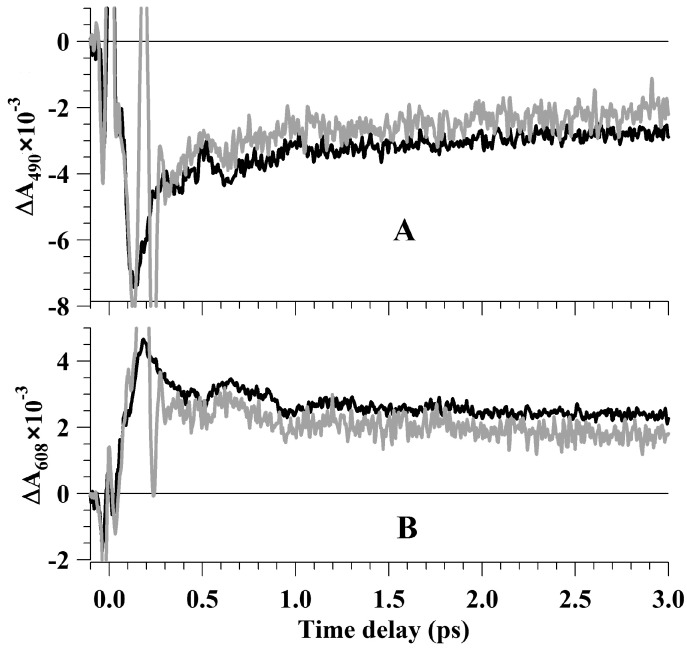
Kinetic curves of photo-induced rhodopsin absorption. (**A**) Probe at 490 nm. (**B**) Probe at 608 nm. Black kinetic curves were recorded after the action of one 25-fs *pump1* at 500 nm. Grey curves were recorded after consecutive action of 25-fs *pump1* at 500 nm and30-fs *pump2* at 620 nm following one another with a 200-fs time delay. These curves were normalized by the time range <0.135 ps (up to the *pump2* occurrence time).

Figure 3 shows the kinetic curves of photo-induced Rh_498_ absorption recorded after the action of one (∆A^1^(t)) and two (∆A^1+2^(t)) pump pulses at 490 and 608 nm probe wavelengths in the time range up to 3 ps. The kinetic curves obtained in both experiments were normalized by a time range <0.135 fs, *i.e.*, prior to the action of *pump2* supplied with a 200-fs time delay. This allowed observation of the direct *pump2* effect on the dynamics of photo-induced Rh_498_ reactions. At times t < 135 fs, the kinetic curves ∆A^1^(t) and ∆A^1+2^(t) were nearly the same, whereas at times t > 300 fs, the optical densities of kinetic curves ∆A^1^(t) and ∆A^1+2^(t) were different (Figure 3A,B). At a time delay of *pump1* of 135–300 fs, the signal ∆A^1+2^(t) was perturbed by a femtosecond laser artifact (Figure 3). The rate of kinetic curves ∆A^1+2^(t) changed compared with ∆A^1^(t) at 300–400 fs after the *pump1* effect and 100–200 fs after the *pump2* effect, respectively. At later times, t > 400–500 fs, the shapes of kinetic curves ∆A^1+2^(t) were similar to ∆A^1^(t) curves, but with just higher optical densities in the Rh_498_ bleaching range (Figure 3A) and with lower optical densities in the range of Photo_570_ and Batho_535_ absorption (Figure 3B). Since the main differences in the ∆A^1^(t) and ∆A^1+2^(t) curves are observed at a time delay of 100–200 fs after *pump2*, one may conclude that all processes of photoexcitation energy transfer from S_1_ to S_0_potential energy surfaces (PES) during the reverse rhodopsin photoreaction are completed by the 200th fs after its initiation by *pump2*. It may be assumed therefore that the excited state Photo_570_ lifetime, *i.e.*, the lifetime of vibrationally excited bathorhodopsin, is comparable with the excited state Rh_498_ lifetime. This correlates well with the theoretical results [19,36], showing that the lifetime of the bathorhodopsin excited state is shorter than for rhodopsin.

**Figure 4:**
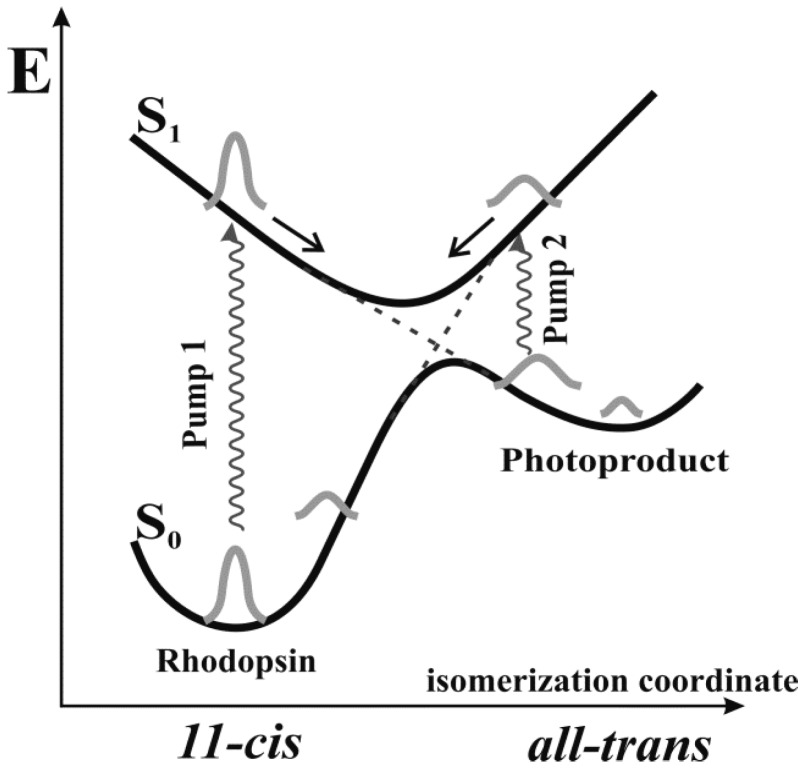
The scheme of the potential energy surfaces of the visual pigment rhodopsin explaining the principle of its photochromism.

After *pump2* effect at time delay t > 400–500 fs and the probe wavelength of 490 nm, the change of optical density was about 0.7 × 10^−3^ relative units (Figure 3A), whereas at the probe wavelength of 608 nm, was is about 0.6 × 10^−3^–1.1 × 10^−3^ relative units (Figure 3B). The observed differences in the characteristics of the kinetic curves with one (*pump1*) and two (*pump1* and *pump2*) pump pulses may be interpreted as a transition of a part of the Photo_570_ molecules back to Rh_498_. For the time of Photo_570_ formation (200 fs), changes in the nearest protein environment of retinal may be neglected. The possibility of reverse photoreaction may be schematically represented using a model of two states, which is shown in Figure 4. Recent model studies on rhodopsin and bathorhodopsin confirm such a structure of the PES for the rhodopsin molecule [19,37]. These studies demonstrated the presence of barrierless PES in bathorhodopsin excited state S_1_ and the same S_1_/S_0_ intersection space for conical intersections (CIs) of rhodopsin and bathorhodopsin. The coherent wave packet formed under the effect of *pump1* moves by the PES of the excited state S_1_. Near the area of CI of the PES (S_1_/S_0_), the wave packet splits into two subpackets jumping to the PES of the electronic ground state S_0_ with formation of Photo_570_ (65%) and Rh_498_ (35%).

Depending on the time delay, *pump2* affects the reaction products: Photo_570_ or Batho_535_. Meanwhile, a wave packet is formed in the right branch of the electron excited state S_1_ (Figure 4). It moves by the PES of S_1_ in the area of CI, most likely also splitting into two subpackets, one of which jumps to the PES of S_0_, forming Rh_498_, which contains 11-*cis* retinal, and the other packet returns to the PES of S_0_of Photo_570_ with non-isomerized *trans*-retinal. This, of course, is just a qualitative explanation of the photochromism observed. However, the implementation of photochromism in the femto- and pico-second range shows that primary photoreactions of rhodopsin really proceed within two PESs.

Photochromic switching efficiency was determined as a part of Rh_498_ molecules returned to the ground state after *pump2* action. Calculations have shown that the quantum yield of the reverse rhodopsin reaction is 14.2% for the Photo_570_→Rh_498_ transition and 16.4% for the Batho_535_→Rh_498_ transition. Previously, the attempts to assess the quantum yield of the reverse rhodopsin photoreaction from bathorhodopsin have been made, both theoretically [36] and experimentally at 77 K [38]. In both cases, values of about 0.5 were obtained, which were approximately three-times greater than in this work.

### 2.3. Coherent Control of Primary Ground-State Rhodopsin Photoproduct Phototransition to Rhodopsin

Oscillations observed in the kinetic curves of photo-induced chromophore isomerization in Rh_498_ reflect the dynamics of the Photo_570_ coherent wave packet. To put it differently, these oscillations reflect the occurrence of quantum interference during the reaction, which results in the time dependence of different stationary vibrational states. It is expected that the reverse phototransition, Photo_570_→Rh_498_, efficiency may significantly depend upon whether *pump2* is absorbed in the maximum or in the minimum of oscillations.

Experiments in which the time delay between *pump1* and *pump2* varied so that the time of *pump2*'s arrival was synchronized with the oscillation phase presented in absorption signals of Photo_570_ were performed. The maximum of the differential absorption spectrum that reflects the formation of Batho_535_ registered 100 ps after *pump1* was assumed as a measure of reverse photoreaction efficiency (Figure 2, black curve).

**Figure 5:**
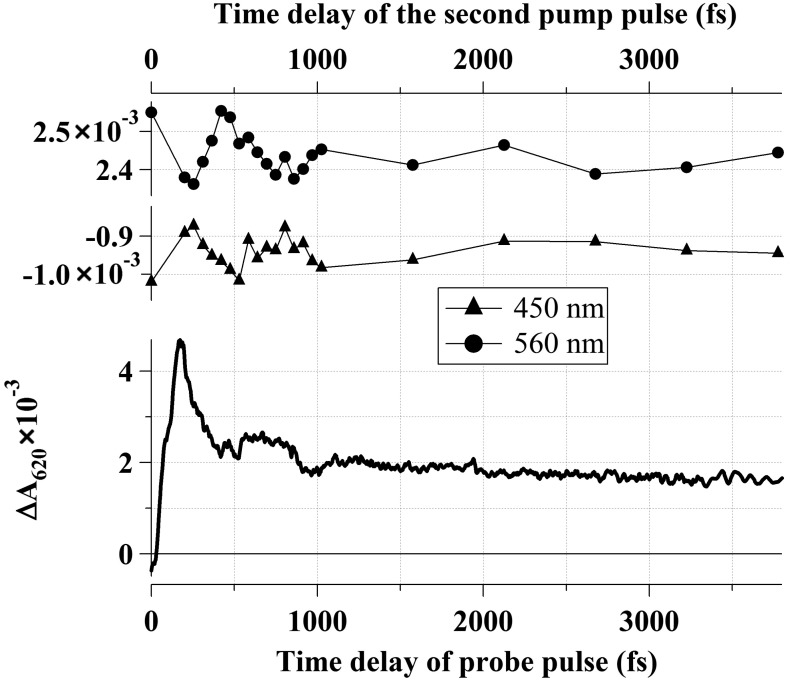
Coherent control of primary ground-state rhodopsin photoproduct phototransition to rhodopsin. (**Bottom**) Kinetic curve of photo-induced rhodopsin absorption under the effect of the 25-fs *pump1* at 500 nm and probing at 620 nm. (**Top**) Variation of photo-induced rhodopsin absorption recorded at probe wavelengths of 450 nm (triangles) and 560 nm (circles) 100 ps after *pump1* and *pump2* excitation at different *pump2* time delays (upper abscissa axis). Optical density at a 0-fs time delay was obtained by exclusive rhodopsin excitation by *pump1* and is assumed as a control.

Figure 5 (at the bottom) shows the kinetic curve of photo-induced Rh_498_ absorption obtained in the experiment with the 25-fs *pump1* at 500 nm, when probed at 620 nm. Figure 5 (at the top) shows values of optical density reflecting variations in the absorption of Batho_535_ at 560 nm (circles) and Rh_498_ at 450 nm (triangles) with respect to the *pump2* time delay. Optical densities at 450 and 560 nm in the differential spectrum observed 100 ps after the exclusive action of *pump1* were taken as a control (Figure 2, black curve). These control values are shown in Figure 5 (at the top) with a 0-fs time delay. The results obtained show that the number of Photo_570_ molecules participating in the reverse photoreaction depends on the synchronization of the *pump2* time delay with phase characteristics of the wave packet in Photo_570_. In the case when *pump2* arrives at the moment of maximum Photo_570_ absorption (200-fs time delay), the reverse photoreaction obtains maximum efficiency. Hence, the differential spectrum recorded 100 ps after excitation (Figure 2, grey curve) shows the maximum signal decrease at the 560-nm wavelength and the maximum signal increase at 450 nm. Photochromic switching is lower when the control pulse (*pump2*) arrives at the antiphase of the coherent vibrational wave packet motion and is absent in the oscillation minimum at a 475-fs time delay (Figure 5, at the top). Similar results were obtained in a broad probing spectral range (420–620 nm). Photochromic switching was observed up to a 4-ps time delay of *pump2*.

Variation of the reverse rhodopsin photoreaction efficiency, observed in this work, which depends on the *pump2* arrival time at different phases of coherent wave packet oscillations, is due to absorption modulation at the S_1_→S_0_ transition by coherent wave packet oscillations in Photo_570_. This is shown by the oscillatory kinetic curve in Figure 1B. Therefore, *pump2* will be absorbed with a higher probability at the maximum of Photo_570_ optical density oscillations. This effect of the photoreversible rhodopsin reaction control by means of the *pump2* action at different oscillation phases of the wave packet might be considered as a coherent control method.

As shown in the model study [19], the quantum yield of both direct and reverse rhodopsin photoreactions depends on the HOOP oscillation phase and the amplitude of the excited molecule. Based on the results of that work, one may suggest the use of two approaches simultaneously: the coherent control demonstrated in this work and the shaped pulses, which affect the HOOP oscillation phase and the quantum yield of the reverse photoreaction, respectively, will increase the photoswitching efficiency. This is critical to the creation of molecular photoswitches.

## 3. Experimental Section

### 3.1. Chemicals

The chemicals used in the experiments were of the highest purity and commercially available. Reagents were purchased from Sigma (St. Louis, MO, USA), Fluka (Buchs, Switzerland), Anatrace (Maumee, OH, USA), Millex and Amicon.

### *3.2.* Rhodopsin Preparation

Fresh bovine eyes (*Bos taurus*) were purchased from a meat processing factory (“Ramensky trade house”, Krasnoarmeiskaya st.131, 140109, Ramenskoe, Moscow region, Russia). The work was done with the permission of the slaughterhouse to use these bovine eyes for scientific research.

Rod outer segments (ROS) from bovine retinas and rhodopsin extracts were prepared by the modified method of Okada *et al.* [39]. Retinas were isolated no later than 3 h after slaughtering, and then 50% sucrose solution in Buffer A (10 mM Mops, pH 7.5, containing 30 mM NaCl, 60 mM KCl, 2 mM MgCl_2_, 0.1 mM phenylmethylsulfonyl fluoride (PMSF), 1 mM dithiothreitol and 0.01% NaN_3_) was added to the retina preparations, 1 mL per each retina. The suspension was vigorously shaken for 3 min and centrifuged for 40 min at 4 °C and 2000*×* *g*. The supernatant was diluted four times with Buffer A and centrifuged for 60 min at 4 °C and 2000*×* *g*. The pellet was resuspended in 25 mL of 40% sucrose in Buffer A, and then 10 mL of Buffer A were layered on top to make a stepwise gradient of density following centrifugation for 60 min at 4 °C and 25,000*×* *g* on the Beckman Coulter Avanti J30I centrifuge equipped with the JS 24,38 rotor. The ROS fraction was sampled at the buffer-sucrose border, diluted with Buffer B (5 mM Tris-HCl, pH 8.0, containing 0.5 mM MgCl_2_, 0.4 mM EDTA, 1 mM dithiothreitol and 0.01% NaN_3_) to a density of 1.05 g/cm^3^. ROS were precipitated by centrifugation for 30 min at 4 °C and 41,400*×* *g*. The pellet was resuspended in distilled water with 0.01% NaN_3_, incubated for 30 min at 20 °C with mixing and centrifuged for 30 min at 4 °C and 41,400*×* *g*. The pellet, consisting of photoreceptor membrane disks, was resuspended in 1.6% *n*-heptyl-β-d-thioglucoside (HTG) detergent in buffer C (0.1 M CH_3_COONa, 0.1 M (CH_3_COO)_2_Zn and 0.01% NaN_3_, pH 6.0), 0.8 mL of the solution per 1 mg of rhodopsin. The extract was incubated for 3 h at 20 °C and then 12 h at 4 °C. The rhodopsin extract was centrifuged for 30 min at 4 °C and 41,400× *g* followed by filtration through a Millipore filter (Millex GS PVDF 0.22 μm) and concentrated using a Millipore centrifuge filter (Amicon Ultra-4 Ultracell 30k). The thusly prepared rhodopsin HTG extracts had a concentration of 4–5 mg/mL and possessed *A*_280_/*A*_500_ = 1.7–1.9 purity. A 2 M solution of NH_2_OH was added to the final concentration of 0.1 M before measurements. All manipulations with samples containing rhodopsin were carried out under dim red illumination.

### 3.3. Femtosecond Spectroscopy Setup

#### 3.3.1. One-Pump Probe Pulse Setup

**Figure 6:**
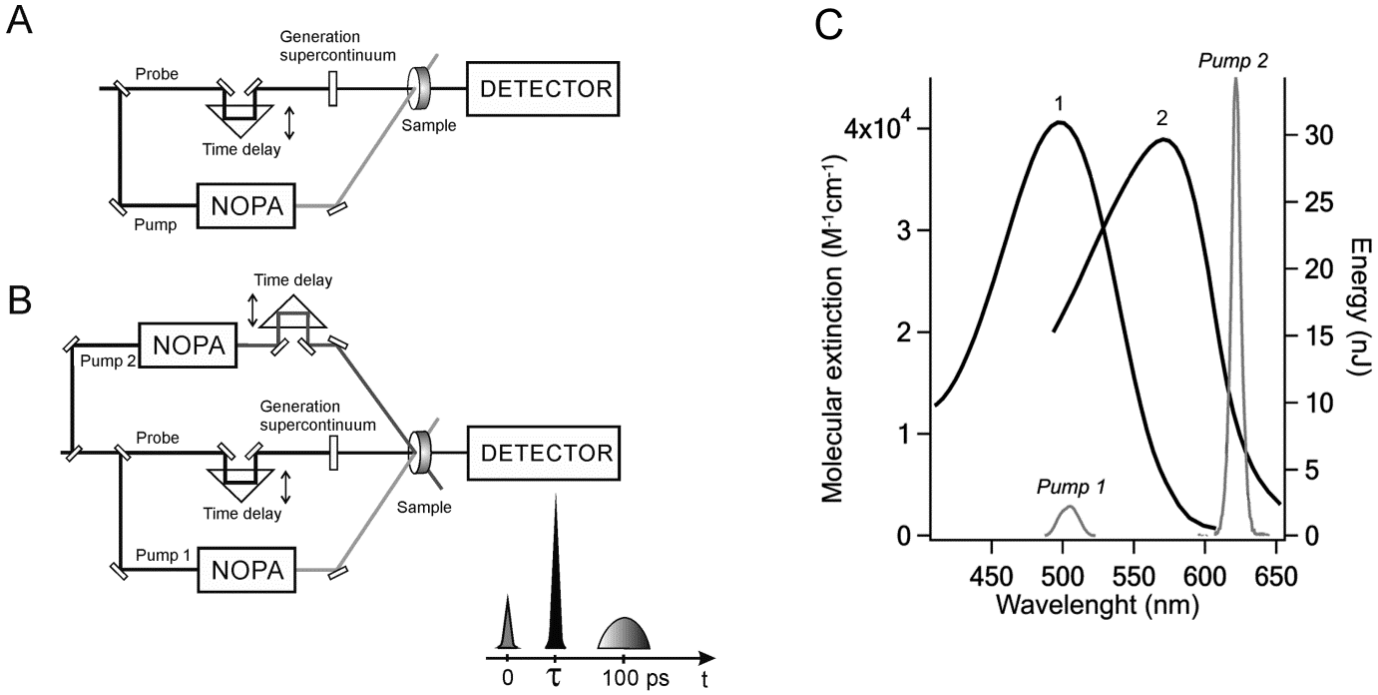
Scheme of the experiments. (**A**) One-pump probe pulse setup. (**B**) Two-pump probe pulse setup. (**C**) Absorption spectra of rhodopsin (1) and primary ground-state rhodopsin photoproduct (2) (spectral data were taken from Kandori *et al.* [6]; black curves, left scale); spectral characteristics of two pump pulses (grey curves, right scale).

Transient absorption spectra were measured by the femtosecond pump-supercontinuum probe setup [40] (Figure 6A). The first pump pulse (*pump1*) was performed by the Gauss pulses with a repetition frequency of 60 Hz, a time duration of 25 fs, a wavelength of 500 nm and energy of 100 nJ. The pump light spot has a diameter of 300 μm. The white supercontinuum pulse was generated in a quartz cell with H_2_O and used as a probe pulse. The diameter of the probe spot was 140 μm. The relative polarization of *pump1* and the probe beam was adjusted to the 54.7° (magic angle) configuration. After the sample, the supercontinuum was dispersed by a polychromator (“Acton SP-300”) and detected by a CCD camera (“Roper Scientific SPEC-10”). Absorption difference spectra ΔA(t, λ) were recorded over the spectral range of 400–740 nm. The measured spectra were corrected for group delay dispersion of the supercontinuum using the procedure described previously [40].

#### 3.3.2. Two-Pump Probe Pulse Setup

For the investigation of the reverse photoreaction and the coherent mechanism of direct rhodopsin photoreaction, two pump pulses and a supercontinuum probe pulse were used (Figure 6B) [12]. The first pump pulse (*pump1*) was described earlier. The second pump pulse (*pump2*) was also generated by a non-collinear phase-matched optical parametric amplifier. It had a wavelength of 620 nm, energy of 700 nJ, a time duration of 30 fs and a light spot of 180 mm in diameter. Both pump pulses were polarized in parallel with one another, but the probe pulse was linearly polarized and rotated by a magic angle relative to the pumps. *Pump2*’s spectral features have been chosen so that they do not overlap the rhodopsin absorption spectrum (Figure 6C). Ultrashort pulses were characterized by spectral phase interferometry for direct electric-field reconstruction (SPIDER). Within the accuracy limits, SPIDER-devised phase instability of the pulses replicas was not observed.

Time delay of *pump2* was chosen in accordance with the phase of the coherent oscillatory packet of the primary rhodopsin photoproduct induced by *pump1* and amounts to 200–1025 fs (steps of 55 fs) and 1025–3750 fs (steps of 550 fs). The probe pulse of supercontinuum was supplied with a time delay of 0.3–10 ps with a step of 3.3 fs and the accumulation of the signal from 50 spectra. A time delay of 100 ps with the accumulation of signals from 10,000 spectra was also used in this work. At this time delay, a differential absorption spectrum only consisting of the absorption band of direct rhodopsin photoreaction product, bathorhodopsin and the rhodopsin bleaching band is registered in the probe spectral range of 400–740 nm. This gives a possibility to monitor the depletion of bathorhodopsin induced by *pump2* as a function of the *pump1–pump2* time delay. This depletion of bathorhodopsin served as a measure of the reverse photoreaction efficiency (the “photochromic switching”).

All experiments were carried out at 21 °C in a 0.2-mm flow optical cell with optical windows of 0.1-mm thickness. The flow rate was such that, after each laser pulse, the absorbing sample was completely refreshed. As a control, the spectra and kinetic curves of photo-induced absorption by similar rhodopsin extract after its complete bleaching were also registered.

### 3.4. Calculation of Quantum Yield for Photo-Induced Transition of Primary Ground-State Rhodopsin Photoproduct to Rhodopsin

The quantum yield of the reverse reaction φ_2_ = N_2_/N_1_^*^, where *N_2_* is the number of molecules of the first photoproduct, Photo_570_, transformed to Rh_498_ by reverse photoreaction and N_1_^*^ is the number of molecules excited by *pump2*, was calculated from the experimental data of the direct Rh_498_→Photo_570_ and reverse Photo_570_→ Rh_498_ phototransitions.

The number of Photo_570_ molecules (*N_1_*) and Batho_535_ molecules, respectively, formed by direct photoreaction may be related to the value of photo-induced Rh_498_ absorption (*∆A^1^_560_*) recorded after *pump1* action at a 560-nm wavelength with a time delay of 100 ps using the following equation: *∆A^1^_560_ =* (*ε_BATHO560_ − ε_RH560_*)*l* *N_1_/N_A_V* = *k_0_N_1_*, where *ε_BATHO560_* and *ε_RH560_* are extinction coefficients for Batho_535_ and Rh_498_ at a 560-nm wavelength [6], *l* is the path length, *N_A_* is the Avogadro constant (6.02 × 10^23^) and *V* is the sample volume subject to *pump1*. Then, *N_1_ = ∆A^1^_560_/k_0_.*

The number of molecules of the first photoproduct transferred to Rh_498_ (*N_2_*) by reverse photoreaction may be expressed via the value of photo-induced Rh_498_ absorption loss (*∆A_560_ = ∆A^1^_560_ − ∆A^1+2^_560_*) in the differential spectrum after *pump2* action at a 560-nm wavelength with a 100-ps time delay, using the same factor k_0_ as follows: *∆A_560_ = k_0_·N_2_*, then *N_2_ = ∆A_560_/k_0_*.

Based on the above expressions, the quantum yield of the reverse rhodopsin photoreaction φ_2_ = N_2_/N_1_^*^ may be represented as follows: *φ_2_ = ∆A_560_/(∆A^1^_560_·P)*, where *P* is the probability of *pump2* absorption to be estimated as follows: *P = σ·Φ*, where *σ* is a cross-section of the first photoproduct molecule absorption; *Φ* is the density of photon flow in *pump2*.

The molecule absorption cross-section of the first photoproduct, *σ_620_*, at the *pump2* wavelength (620 nm) was calculated as follows: *σ_620_* = 3.826 × 10^−21^·*ε_620_*, where *ε*_620_(Photo) = 12,485 M^−1^·cm^−1^ [6]. Thus, *σ_620_*(Photo) = 4.77 × 10^−17^ cm^2^.

Photon flux density (*Ф*) in *pump2* was calculated as follows: *Ф = N*/*S*, where *N* is the number of photons in *pump2*; *S* is the area of the cuvette with the sample affected by the pulse.

The number of photons in *pump2* (*N*) was calculated from the following expression: *N* = *E_pump2_*/*E_ph_*, where *E_pump2_* is the *pump2* energy (7 × 10^−7^ J in this experiment) and *E_ph_* is the photon energy with the wavelength λ = 620 nm (*E_ph_* = 3.2039 × 10^−19^ J). Thus, the number of photons in *pump2* amounts to *N* = 2.1848 × 10^12^.

In the experiment, the area of the sample cuvette affected by *pump2* is *S* = 2.5447 × 10^−4^ cm^2^; as a consequence, the photon flux density is *Ф* = 8.5857 × 10^15^ photon/cm^2^; thereby, the probability of the pulse absorption (*P*) is 0.41.

In the experiment, the optical density (*A_1_*) of photo-induced Rh_498_ absorption 100 ps after excitation by *pump1* at a 560-nm wavelength is 0.00258 optical density units. The optical density decrease (*ΔA*) in the differential spectrum of photo-induced Rh_498_ absorption at a 560-nm wavelength, 100 ps after *pump2* action, with a 200-fs time delay, is 0.00015 optical density units. Thus, the quantum yield of reverse Photo_570_→ Rh_498_ phototransition (*φ_2_*) is 14.2%.

The quantum yield of reverse phototransition Batho_535_→ Rh_498_*φ_2_* for a *pump2* time delay of 3.75 ps, when the photon is already absorbed by Batho_535_, was also estimated. It was found equal to 16.4%.

## 4. Conclusions

Thus, this work shows, firstly, phototransitions Rh_498_→Photo_570_→Rh_498_ occurring in a femtosecond scale at room temperature. Secondly, it is shown that photochromic switching efficiency depends on the timing of oscillation maximum for the dynamics of the coherent vibrational wave packet of Photo_570_ and *pump2*. Such dependence is the obvious example of coherent control over the reverse photoreaction of Rh_498_ chromophore group photoisomerization.

The results obtained allow the consideration of the visual pigment rhodopsin as a model for creating an ultrafast molecular photoswitch. The mechanism of rhodopsin photoswitching described in this work may underlay the functionality of an optical logical element probably designed for use in optical information transfer and processing devices with a running speed exceeding that of the existing analogues [16].

As photochromic materials, various retinal-binding proteins can be used: visual pigments rhodopsins from invertebrates and vertebrate animal eyes, sensor rhodopsins and photodependant ionic pumps of microorganisms. The use of various mutant and modified protein forms is also possible, modifications being present in both the peptide part of the molecule and chromophore.

Therefore, searching for artificial matrices for a retinal chromophore capable of simulating the natural protein matrix is possible. There are some examples for retinal and its analogues [15,41,42,43]. The principle of the coherent control of rhodopsin photochromic reactions induced by femtosecond laser pulses at room temperature proposed in this work and some our other studies [9,12,16] may also be found useful for the development of quantum computers.
